# Blood pressure in adults with cerebral palsy: a systematic review and meta-analysis of individual participant data

**DOI:** 10.1097/HJH.0000000000002912

**Published:** 2021-06-07

**Authors:** Suzie Noten, Rita J.G. van den Berg-Emons, Deborah E. Thorpe, Patricia C. Heyn, Christina M. Marciniak, Patrick G. McPhee, Robert P. Lamberts, Nelleke G. Langerak, Olaf Verschuren, Tommi Salokivi, Katherine M. Morrison, Mark D. Peterson, Chonnanid Limsakul, Henk J. Stam, Grigorios Papageorgiou, Jorie Versmissen, Wilma M.A. Van Der Slot

**Affiliations:** a Department of Rehabilitation Medicine, Erasmus MC University Medical Center Rotterdam; b Rijndam Rehabilitation, Rotterdam, the Netherlands; c Division of Physical Therapy, University of North Carolina at Chapel Hill, School of Medicine, Chapel Hill, North Carolina; d Department of Physical Medicine and Rehabilitation, University of Colorado Anschutz Medical Campus; e Center for Gait and Motion Analysis, Children's Hospital Colorado, Aurora, Colorado, USA; f Department of Physical Medicine and Rehabilitation, Northwestern University Feinberg School of Medicine; g Shirley Ryan AbilityLab, Chicago, Illinois, USA; h Department of Pediatrics; i CanChild Centre for Childhood Disability Research; j School of Rehabilitation Science, McMaster University, Hamilton, Ontario, Canada; k Division of Orthopaedic Surgery, Department of Surgical Sciences, Faculty of Medicine and Health Sciences, Stellenbosch University, Tygerberg; l Department of Sport Science, Faculty of Medicine and Health Sciences, Stellenbosch University, Stellenbosch; m Neuroscience Institute and Division of Neurosurgery, Department of Surgery, Faculty of Health Sciences, University of Cape Town, Cape Town, South Africa; n Center of Excellence for Rehabilitation Medicine, UMC Utrecht Brain Center, University Medical Center Utrecht, Utrecht University and De Hoogstraat Rehabilitation, Utrecht, The Netherlands; o Support and Expert Center for Persons with Intellectual Disability, KTO, Paimio, Finland; p Department of Pediatrics, Centre for Metabolism, Obesity and Diabetes Research, McMaster University, Hamilton, Ontario, Canada; q Department of Physical Medicine and Rehabilitation, University of Michigan, Ann Arbor, Michigan, USA; r Department of Rehabilitation Medicine, Faculty of Medicine, Prince of Songkla University, Songkhla, Thailand; s Department of Biostatistics; t Department of Epidemiology; u Departments of Pharmacy and Internal Medicine, Erasmus MC University Medical Center Rotterdam, Rotterdam, the Netherlands

**Keywords:** adults, blood pressure, cerebral palsy, hypertension, meta-analysis, risk factors, systematic review

## Abstract

**Objectives:**

This systematic review and meta-analysis was designed to determine the overall mean blood pressure and prevalence of hypertension among a representative sample of adults living with cerebral palsy by combining individual participant data. Additional objectives included estimating variations between subgroups and investigating potential risk factors for hypertension.

**Methods:**

Potential datasets were identified by literature searches for studies published between January 2000 and November 2017 and by experts in the field. Samples of adults with cerebral palsy (*n* ≥ 10, age ≥ 18 years) were included if blood pressure data, cerebral palsy-related factors (e.g. cerebral palsy subtype), and sociodemographic variables (e.g. age, sex) were available. Hypertension was defined as at least 140/90 mmHg and/or use of antihypertensive medication.

**Results:**

We included data from 11 international cohorts representing 444 adults with cerebral palsy [median (IQR) age of the sample was 29.0 (23.0–38.0); 51% men; 89% spastic type; Gross Motor Function Classification System levels I–V]. Overall mean SBP was 124.9 mmHg [95% confidence interval (CI) 121.7–128.1] and overall mean DBP was 79.9 mmHg (95% CI 77.2–82.5). Overall prevalence of hypertension was 28.7% (95% CI 18.8–39.8%). Subgroup analysis indicated higher blood pressure levels or higher prevalence of hypertension in adults with cerebral palsy above 40 years of age, men, those with spastic cerebral palsy or those who lived in Africa. BMI, resting heart rate and alcohol consumption were risk factors that were associated with blood pressure or hypertension.

**Conclusion:**

Our findings underscore the importance of clinical screening for blood pressure in individuals with cerebral palsy beginning in young adulthood.

## INTRODUCTION

Cerebral palsy is the most common childhood-onset physical disability, with an incidence of 2–3 per 1000 live births [1]. Cerebral palsy is caused by a nonprogressive disturbance to the developing fetal or infant brain that affects movement and posture [2]. The life expectancy of individuals with cerebral palsy has improved in recent decades, and increasing numbers of children with cerebral palsy now survive into adulthood; therefore, it is important to understand the long-term effects of cerebral palsy across the lifespan [3,4].

In adults with cerebral palsy, functional deterioration [5], low levels of aerobic fitness [6,7] and physical activity [8,9], pronounced sedentary behavior [10], and obesity [7,11] are prevalent. From the general population, it is known that these factors are associated with the risk of developing cardiovascular disease (CVD) [12], suggesting that adults with cerebral palsy may be at increased risk. Indeed, in recent years, adults with cerebral palsy have been shown to have a greater risk of CVD than the general population [11–14]. However, the literature is scarce and clinical attention towards CVD risk factors in adults with cerebral palsy is limited. One of the main risk factors of CVD is high blood pressure (BP), which is an important problem worldwide and was the leading cause of death and disability in 2010 [15].

Research and clinical practice have done little to understand BP in people with cerebral palsy, and as a consequence, there is limited knowledge of hypertension risk in this population. To date, only a few studies reported the prevalence of hypertension among adults with cerebral palsy, which ranged between 14 and 30% [7,16–18]. Furthermore, those studies were limited by small sample size, relatively young age [7,16,18], or assessed self-reported hypertension [17]. Therefore, no uniform conclusion on BP levels in cerebral palsy can be drawn from these publications, and reliable hypertension prevalence estimates are not available. In addition, little is known about specific subgroups of adults with cerebral palsy who might be at increased risk (e.g. subtype of cerebral palsy or level of gross motor functioning), as well as potential risk factors influencing BP levels, such as BMI or physical (in)activity. This knowledge would contribute to a better understanding of hypertension risk in adults living with cerebral palsy, which is urgently needed in current clinical practice and future research in this area.

Therefore, we performed a systematic review and meta-analysis, combining individual participant data (IPD) from available published and unpublished studies on BP in adults with cerebral palsy. This study was designed to determine the overall mean level of BP and the prevalence of both prehypertension and hypertension. We also aimed to estimate variations in BP levels and prevalence of prehypertension and hypertension by age, sex and cerebral palsy characteristics and to explore associations between potential risk factors and BP levels (e.g. biological and lifestyle-related risk factors).

## METHODS

This systematic review and meta-analysis followed the guidelines outlined in the Preferred Reporting Items for a Systematic Review and Meta-analysis of Individual Participant Data (PRISMA-IPD Statement) [19]. The study was approved by the Medical Ethical Committee of the Erasmus MC University Medical Center, Rotterdam, The Netherlands (MEC-2017-1084).

### Study selection process

A systematic literature search in Embase, Medline Ovid, PsycINFO Ovid, CINAHL, Cochrane, Web of Science and Google Scholar databases was performed for studies published between January 2000 and November 2017, with the following broad search terms: blood pressure or hypertension and cerebral palsy. The detailed search strategy was developed in consultation with an information specialist and considered only full-text articles without any language constraints (Data Supplement S1).

Results from the different databases were combined and duplicates removed. First, two independent reviewers (S.N. and C.L.) screened titles and abstracts for eligibility; full-text articles were obtained from potentially eligible articles and screened. Subsequently, the results of both reviewers were compared, and differences were discussed in a consensus meeting. When consensus could not be reached, a third reviewer (R.v.d.B.-E.) was consulted. References of included studies, as well as conference proceedings, were checked to further identify potentially relevant studies. To identify unpublished studies, experts working in the field were approached to inquire for potential datasets.

Studies were eligible if they fulfilled the following criteria: observational study or trial (baseline data); study was approved by a Medical Ethical Committee, and informed consent of the study participants was available; recruitment took place in the year 2000 or more recently; sample size was at least 10 adults with cerebral palsy (≥18 years); BP and essential sample characteristics: age, sex and cerebral palsy characteristics (type, distribution or gross motor functioning) were available. Studies were excluded if BP data were self-reported, self-measured at home or measured with a finger or wrist cuff device. In addition, samples with only hypertensive participants were excluded.

### Data collection

The corresponding authors/investigators of eligible studies were contacted to confirm the inclusion criteria and to start the collaboration with an agreement to share anonymous data. Information on study design, method of measurement, BP data, usage of antihypertensive medication, sample characteristics, cerebral palsy-related factors, biological risk factors and lifestyle-related risk factors were requested from the primary investigator. Eligible and anonymous IPD were safely shared using encrypted files and checked for both completeness and correctness. Samples were included up to November 2018. The primary meta-analysis and all sub-analyses were performed in December 2018 to March 2020.

### Methodological quality assessment

Two investigators independently assessed the methodological quality (S.N. and C.L.), using 11 items of the Strengthening the Reporting of Observational Studies in Epidemiology (STROBE) Statement [20], selected in a previous meta-analysis by our research group [21]. Items were scored as yes (1), partially (0.5) or no (0). A study was considered high quality when the total score was eight or more. Disagreements in rating were discussed until consensus was reached. If necessary, a third reviewer was consulted (R.v.B.-E.). The main publication of each included sample was used to score the methodological quality. In the case that documentation in a publication was insufficient, other publications of the same sample were checked if available, or the primary investigator was contacted to provide the missing information; scores were adjusted accordingly. Primary investigators were also contacted in the event that studies had not yet been published; in this case, the study protocol was used to score the methodological quality. All studies were included in the analysis, regardless of their methodological quality scores.

### Data items and determinants

Primary outcomes were overall mean SBP and DBP, and the prevalence of prehypertension and hypertension. Participants without SBP and/or DBP data were excluded. Prevalence of prehypertension and hypertension was defined by the hypertension guidelines of the European Society of Cardiology and European Society of Hypertension [12]. In the guidelines, prehypertension is defined as SBP 130–139 mmHg and/or DBP 85–89 mmHg, and hypertension as SBP at least 140 mmHg and/or DBP at least 90 mmHg, or use of antihypertensive medication. These guidelines were used as they were comparable with the previous American Hypertension guidelines [22], which were applicable in the period in which the included studies were performed.

Classification of BP was evaluated as determined by the European Hypertension guidelines. BP was classified as optimal (SBP <120 mmHg and DBP <80 mmHg), normal (SBP 120–129 mmHg and/or DBP 80–84 mmHg), high normal (SBP 130–139 mmHg and/or DBP 85–89 mmHg), grade 1 hypertension (SBP 140–159 mmHg and/or DBP 90–99 mmHg), grade 2 hypertension (SBP 160–179 mmHg and/or DBP 100–109 mmHg) or grade 3 hypertension (SBP ≥180 mmHg and/or DBP ≥110 mmHg).

Personal characteristics were obtained if available, and included: intellectual disability (defined as a moderate-to-severe level of intellectual functioning, indicated as an IQ level below 70) [23], level of education, employment, civil status and living situation.

Subgroups of adults with cerebral palsy were categorized by age, sex, cerebral palsy subtype, cerebral palsy distribution, GMFCS level and continent of residence, to estimate the effect of each of these factors on BP levels and prevalence of prehypertension and hypertension. Age was classified into three categories (18–29, 30–39 and ≥40 years). Cerebral palsy subtype was classified according to neurological signs [spastic or other subtypes (dyskinetic, ataxic or mixed)] and distribution to unilateral or bilateral [24]. Gross motor functioning was classified using the Gross Motor Function Classification System (GMFCS) [25]. Continents of the included samples were North America, Europe and Africa. Reference groups can be found in Table 3.

If available, the following data were collected from the original authors to explore the effect of potential risk factors on BP levels and hypertension: cerebral palsy-related factors (muscle tone, pain, fatigue), biological risk factors [family history of CVD, BMI, waist-to-hip ratio, resting heart rate, aerobic fitness, total cholesterol (TC), high-density lipoprotein cholesterol (HDL), low-density lipoprotein cholesterol (LDL), TC/HDL ratio, triglycerides, glucose, insulin and diabetes] and lifestyle-related risk factors (alcohol consumption, smoking and physical activity). Data on the following factors were limited and could not be included in the multivariable analysis, thus only descriptive results are reported: cerebral palsy-related factors of pain and fatigue, biological risk factors waist-to-hip ratio, aerobic fitness, TC, HDL, LDL, TC/HDL ratio, triglycerides, glucose, insulin and diabetes and lifestyle-related risk factor physical activity. In case the scaling or type of measurement differed across datasets, variables were translated to common scales if possible. Conversion to common scales or outcomes measures was needed for intellectual disability, muscle tone, pain, fatigue, aerobic fitness and physical activity. Data Supplement S2 provides a description of all procedures of translating variables to ensure common scales across studies [26–32].

The American Hypertension guidelines proposed by the American College of Cardiology and the American Heart Association were recently adapted and lower cut-off values for prehypertension and hypertension were recommended [33]. The impact of this change on the prevalence of prehypertension and hypertension was explored in this study. Prehypertension was determined by the new guidelines as SBP 120–129 mmHg and DBP less than 80 mmHg; hypertension as SBP at least 130 mmHg or DBP at least 80 mmHg or use of antihypertensive medication. We also evaluated classification of BP as determined by the new guidelines; BP was classified as normal (SBP <120 mmHg and DBP <80 mmHg), elevated (SBP 120–129 mmHg and DBP <80 mmHg), hypertension stage 1 (SBP 130–139 mmHg or DBP 80–89 mmHg), hypertension stage 2 (SBP 140–180 mmHg or DBP 90–120 mmHg) or hypertensive crisis (SBP >180 mmHg and/or DBP >120 mmHg).

### Statistics

Descriptive statistics were performed for personal characteristics, cerebral palsy-related factors, biological and lifestyle-related risk factors. In case of more than one BP measurement in a person, the median BP was used for analysis; in case of only one measurement, this measurement was used.

#### Primary analyses

Estimates and 95% CI for the primary outcomes were obtained by a two-stage meta-analysis model. First, the means and standard errors of SBP and DBP and proportions and standard errors of prehypertension and hypertension were estimated from the IPD. Secondly, pooled estimates for the outcomes were obtained via a random-effects meta-analysis model using the DerSimonian and Laird estimator [34] and the arcsine transformation (for proportions) [35]. The random-effects model takes the heterogeneity of samples into account. Statistical heterogeneity was quantified using the *I*^*2*^ measure, which describes the amount of variation attributed to heterogeneity rather than sampling error across samples [36]. Funnel plots for BP were created to inspect for evidence of publication bias. Descriptive statistics were used to explore the classification of BP.

#### Secondary analyses

Linear and logistic multivariable regression models, including study as a fixed-effect were used to estimate the association of age, sex, cerebral palsy subtype, cerebral palsy distribution, GMFCS level and continent with the primary outcomes. Estimates were adjusted for these factors. Estimated beta coefficients (*β*) and odds ratios (ORs) and 95% CIs were calculated. A *P* value 0.05 or less was considered significant.

To explore the effect of potential risk factors on BP levels, separate extended multivariable regression models were used, adjusted for one risk factor each. This method was performed as a multivariable regression model including all the factors was not feasible because of the large number of parameters to be estimated. These risk factors were selected based on availability of the data and included muscle tone, family history of CVD, BMI, resting heart rate, alcohol consumption and smoking. The models were adjusted for age, sex, cerebral palsy subtype, cerebral palsy distribution, GMFCS level and continent. *P* values were adjusted for multiple comparisons using the Holm method.

Use of antihypertensive medication was used to define hypertension. If the information on the use of antihypertensive medication was missing, and BP levels were normal, hypertension could not be defined, and the participant was excluded from the analysis. Participants using antihypertensive medication or who had missing information on the use of antihypertensive medication were excluded from the analyses to determine the overall mean SBP and DBP and prehypertension, and subgroup analyses.

## RESULTS

### Study selection and characteristics

The literature review produced a total of 1144 potentially eligible articles after removal of duplicates. After title and abstract screening, 41 full-text articles were reviewed, and seven published studies were found eligible. In addition, 31 experts in the field were approached to obtain unpublished studies, which resulted in an additional 11 eligible studies. Most excluded studies had clinically based BP data obtained from medical records or registers. These data were collected during regular medical checks or hospitalization, and might have been affected by other illnesses or procedures (e.g. surgery); also, it was suggested by the contact person that sample characteristics were often limited or unavailable.

**FIGURE 1:**
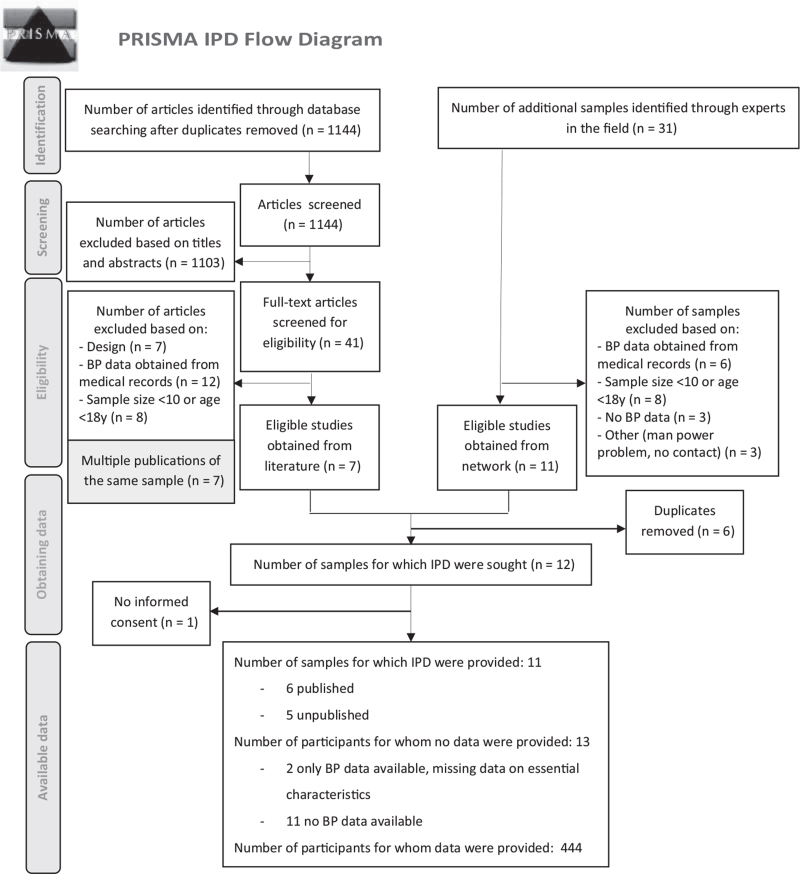
The PRISMA-IPD flow diagram.

Six duplicates were found and removed, and one study was excluded as no informed consents were available. Eventually, 11 studies (six published, five unpublished at that time) met the inclusion criteria [7,13,37–43], Lamberts *et al.*, unpublished data, 2017, Verschuren *et al*., unpublished data, 2015–2016) and all primary investigators agreed to collaborate (Fig. 1). Included studies were five cross-sectional studies [7,37,40,42], Verschuren *et al.*, unpublished data, 2015–2016 five cohort studies [13,38,39,43], Lamberts *et al.*, unpublished data, 2017, and one RCT (baseline measurement was used) [41], executed between 2004 and 2017 in North America, Europe and Africa.

A total of 444 adults with cerebral palsy, 51% men, mainly with spastic cerebral palsy (89%) and GMFCS levels I–V were included. Median (IQR) age of the sample was 29.0 (23.0–38.0). Thirty-seven participants (8%) used antihypertensive medication, and four participants (1%) had missing information on the use of antihypertensive medication (Table 1). Personal characteristics, cerebral palsy-related factors, biological and lifestyle-related risk factors are presented in Data Supplement S3.

**TABLE 1:** Study characteristics of the 11 single studies

				Sample characteristics						
Study	Country	Design	Year of assessment	*n*	Age median (IQR)	Sex [*n* (%)]	CP subtype [*n* (%)]	CP distribution [*n* (%)]	GMFCS level [*n* (%)]	Number of participants using antihypertensive medication [*n* (%)]
Total sample				444	29.0 (23.0–38.0)	M: 228 (51)F: 216 (49)	Spas: 313 (89)Other: 40 (11)Md: 91	Uni: 97 (23)Bi: 320 (77)Md: 27	I: 140 (33)II: 139 (32)III: 73 (17)IV: 53 (12)V: 27 (6)Md: 12	37 (8)Md: 4
Heyn *et al*. [13]	USA, Colorado	Cohort (baseline)	2015–2017	70	23.9 (21.1–27.4)	M: 32 (46)F: 38 (54)	md	Uni: 26 (37)Bi: 44 (63)	I: 28 (40)II: 27 (38)III: 13 (19)IV: 2 (3)V: 0 (0)	2 (3)
Marciniak *et al*. [42]	USA, Illinois	Cross-sectional	2014–2017	46	29.5 (25.0–43.0)	M: 19 (41)F: 27 (59)	Spas: 12 (32)Other: 25 (68)Md: 9	Uni: 5 (11)Bi: 41 (89)	I: 7 (15)II: 8 (17)III: 9 (20)IV: 15 (33)V: 7 (15)	6 (13)
Thorpe *et al*. [38]	USA, North Carolina	Cohort (baseline)	2006–2012	89	27.0 (23.0–36.0)	M: 47 (53)F: 42 (47)	Spas: 89 (100)Other: 0 (0)	Uni: 28 (31)Bi: 61 (69)	I: 30 (34)II: 22 (25)III: 16 (18)IV: 18 (20)V: 3 (3)	15 (17)
McPhee *et al.*[40]	Canada, Ontario	Cross- sectional	2012–2014	42	31.0 (24.2–37.8)	M: 21 (50)F: 21 (50)	Spas: 31 (74)Other: 11 (26)	Uni: 10 (26)Bi: 29 (74)Md: 3	I: 5 (12)II: 9 (21)III: 10 (24)IV: 11 (26)V: 7 (17)	0 (0)
Morrison *et al.*[39]	Canada, Ontario	Cohort	2011–2013	12	33.0 (31.0–34.0)	M: 6 (50)F: 6 (50)	md	Uni: 6 (50)Bi: 6 (50)	md	2 (17)
van den Berg-Emons *et al*. [41]	The Netherlands	RCT (baseline)	2010	44	21.0 (19.0–22.0)	M: 23 (52)F: 21 (48)	Spas: 44 (100)Other: 0 (0)	Uni: 21 (49)Bi: 22 (51)Md: 1	I: 25 (57)II: 16 (37)III: 2 (4)IV: 1 (2)V: 0 (0)	0 (0)
van der Slot *et al*. [7]	The Netherlands	Cross- sectional	2004–2006	51	36.0 (32.5–41.5)	M: 33 (65)F: 18 (35)	Spas: 51 (100)Other: 0 (0)	Uni: 0 (0)Bi: 51 (100)	I: 12 (23)II: 26 (51)III: 9 (18)IV: 4 (8)V: 0 (0)	2 (4)
Verschuren *et al*. unpublished data, 2015–2016	The Netherlands	Cross- sectional	2015–2016	23	34.8 (22.9 – 50.9)	M: 12 (52)F: 11 (48)	Spas: 23 (100)Other: 0 (0)	Md	I: 9 (39)II: 8 (35)III: 4 (18)IV: 1 (4)V: 1 (4)	1 (4)Md: 4
Salokivi *et al*. [37]	Finland	Cross- sectional	2013–2014	14	40.0 (26.0–46.5)	M: 7 (50)F: 7 (50)	Spas: 10 (71)Other: 4 (29)	Uni: 1 (7)Bi: 13 (93)	I: 0 (0)II: 2 (14)III: 2 (14)IV: 1 (7)V: 9 (65)	0 (0)
Lamberts *et al*., unpublished data, 2017	South Africa	Cohort (baseline)	2017	28	38.9 (34.6–45.6)	M: 12 (43)F: 16 (57)	Spas: 28 (100)Other: 0 (0)	Uni: 0 (0)Bi: 28 (100)	I: 11 (39)II: 12 (43)III: 5 (18)IV: 0 (0)V: 0 (0)	5 (18)
Langerak *et al.*[43]	South Africa	Cohort (baseline)	2017	25	35.9 (34.3–41.1)	M: 16 (64)F: 9 (36)	Spas: 25 (100)Other: 0 (0)	Uni: 0 (0)Bi: 25 (100)	I: 13 (52)II: 9 (36)III: 3 (12)IV: 0 (0)V: 0 (0)	4 (16)

Bi, bilateral; F, female; GMFCS, Gross Motor Function Classification System; M, male; Md, missing data; other, other subtypes of cerebral palsy (dyskinetic, ataxic or mixed); RCT, randomized controlled trial; Spas, spastic; Uni, unilateral.

### Methodological quality assessment

The results of the methodological quality assessment are presented in Data Supplement S4. There was an 83% agreement (73 of 88 items) between the two raters, and disagreements regarding the scoring were minimal and were all solved in a consensus meeting. All studies had good methodological quality (score above 8), except for one study rated as 5 [37].

### Blood pressure

BP was measured between one and five times per participant across studies, of which five studies measured BP once. Measurements were primarily performed with a digital device; in two of the samples, BP was measured manually [7,41]. Devices were properly maintained, calibrated, and validated, and appropriately sized cuffs were used for assessments. Five studies measured BP at the unaffected or least affected side [7,38,39,42], Verschuren *et al.*, unpublished data, 2015–2016 four at the left arm [37,41,43], Lamberts *et al*., unpublished data, 2017, and two at the right arm [13,40] (Data Supplement S5).

**FIGURE 2:**
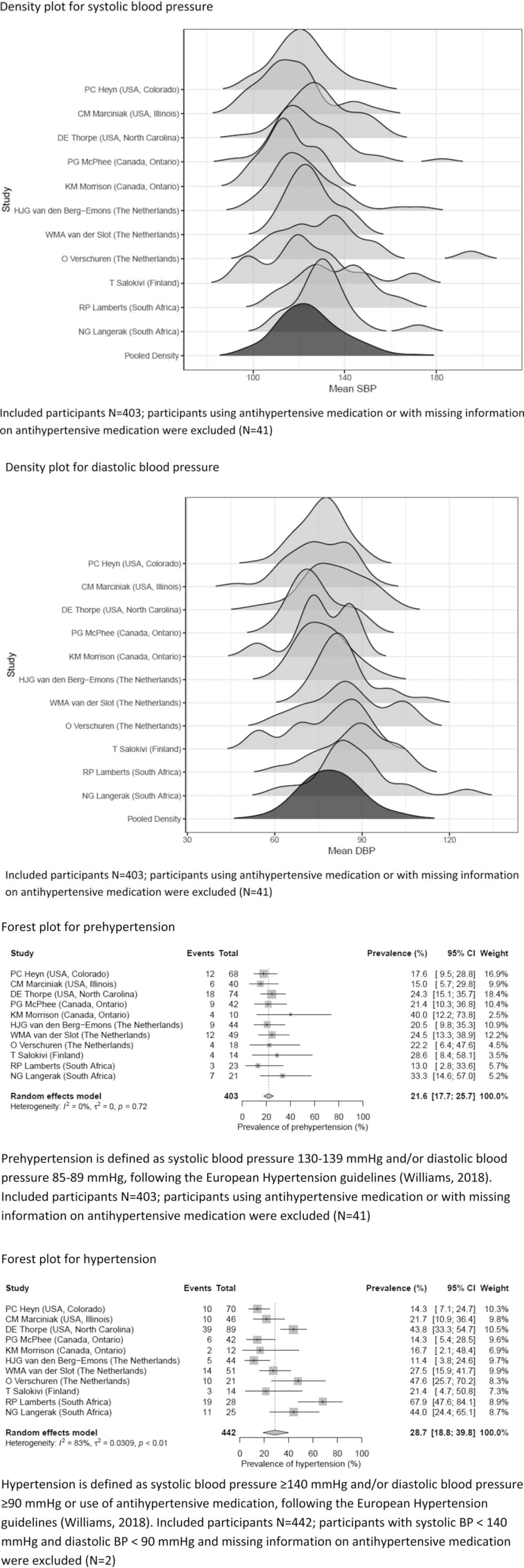
Density plots for SBP and DBP and forest plots for overall prevalence of prehypertension and hypertension.

The overall mean SBP of the total sample was 124.9 mmHg (95% CI 121.7–128.1), and the overall mean DBP was 79.9 mmHg (95% CI 77.2–82.5). According to the European Hypertension guidelines, the overall prevalence of prehypertension was 21.6% (95% CI 17.7–25.7) and the overall prevalence of hypertension was 28.7% (95% CI 18.8–39.8). Density plots for SBP and DBP and forest plots for prehypertension and hypertension can be found in Fig. 2. The level of heterogeneity (*I*^*2*^) was substantial (>75%) for most of the analyses, which reflects considerable variation in results between studies.

The funnel plots for SBP and DBP indicated that publication bias was highly unlikely as there were study effects reported on both sides of the pyramid (Data Supplement S6).

BP levels of more than half of the participants were classified as optimal (30.0%, SBP <120 mmHg and DBP <80 mmHg) or normal (25.8%, SBP 120–129 mmHg and DBP 80-84 mmHg). The number and percentage of participants per classification of BP can be found in Table 2.

**TABLE 2:** Classification of blood pressure following the European Hypertension guidelines

Category	SBP (mmHg)		DBP (mmHg)	Number of participants	Percentage of participants
Optimal	<120	And	<80	121	30.0
Normal	120–129	And/or	80–84	104	25.8
High normal	130–139	And/or	85–89	88	21.8
Grade 1 hypertension	140–159	And/or	90–99	67	16.6
Grade 2 hypertension	160–179	And/or	100–109	18	4.5
Grade 3 hypertension	≥180	And/or	≥110	5	1.3

Data from Williams (2018). Included participants *N* = 403; participants using antihypertensive medication or with missing information on antihypertensive medication were excluded (*N* = 41).

Adults with cerebral palsy above 40 years of age were more likely to have high SBP than adults aged 18–29 (*β* = 5.14, 95% CI 1.04–9.24, *P* = 0.014). Males were more likely to have high SBP than women (*β* = 3.87, 95% CI 0.75–7.00, *P* = 0.015). Lower SBP levels were found in other subtypes of cerebral palsy than in spastic cerebral palsy (*β* = −7.06, 95% CI −12.25 to −1.87, *P* = 0.008). Also, higher SBP levels were found in individuals in Africa when compared with Europe (*β* = 8.35, 95% CI 3.25–13.44, *P* = 0.001). DBP was higher in adults greater than 40 years of age compared with adults aged 18–29 (*β* = 4.02, 95% CI 0.92–7.11, *P* = 0.011). Higher DBP levels were found in individuals in Africa when compared with Europe (*β* = 7.79, 95% CI 3.94–11.64, *P* < 0.001). Prevalence of hypertension was higher in adults above 40 years of age than in adults aged 18–29 (OR = 2.91, 95% CI 1.52–5.62, *P* = 0.001). It was also higher in Africa (OR = 3.90; 95% CI 1.77–8.82, *P* < 0.001) and North America (OR = 2.31, 95% CI 1.23–4.49, *P* = 0.011) compared with Europe. The results of subgroup analyses are presented in Table 3.

**TABLE 3:** Mean SBP and DBP, prehypertension and hypertension per age categories, sex, cerebral palsy subtype, cerebral palsy distribution, Gross Motor Function Classification System and continent

		SBP			DBP		
	*N*	Mean (95% CI)	Beta (95% CI)	*P* value	Mean (95% CI)	Beta (95% CI)	*P* value
Age							
18–29	212	124.7 (122.2–127.1)	Reference category	Reference category	79.6 (77.7–81.4)	Reference category	Reference category
30–39	107	125.7 (122.6–128.7)	1.01 (−3.07–5.10)	0.626	79.9 (77.6–82.2)	0.33 (−2.76–3.41)	0.834
≥40	84	129.8 (126.7–132.9)	5.14 (1.04–9.24)	0.014	83.6 (81.2–85.9)	4.02 (0.92–7.11)	0.011
Sex							
Women	192	124.2 (122.0–126.5)	Reference category	Reference category	79.7 (78.0–81.4)	Reference category	Reference category
Men	211	128.1 (126.0–130.2)	3.87 (0.75–7.00)	0.015	81.6 (80.0–83.2)	1.92 (−0.44–4.27)	0.111
CP subtype							
Spastic	281	127.1 (125.5–128.8)	Reference category	Reference category	81.1 (79.8–82.3)	Reference category	Reference category
Other	37	120.1 (115.3–124.9)	−7.06 (−12.25–−1.87)	0.008	78.3 (74.7–81.9)	−2.75 (−6.67–1.17)	0.168
CP distribution							
Unilateral	91	126.8 (122.7–130.9)	Reference category	Reference category	79.2 (76.1–82.3)	Reference category	Reference category
Bilateral	290	126.2 (124.4–128.0)	−0.65 (−5.38–4.08)	0.786	81.1 (79.7–82.5)	1.92 (−1.65–5.49)	0.291
GMFCS							
I	128	125.6 (122.4–128.7)	Reference Category	Reference Category	80.4 (78.0–82.7)	Reference Category	Reference Category
II	129	128.9 (126.1–131.7)	3.32 (−0.87–7.51)	0.120	83.0 (80.9–85.1)	2.62 (−0.55–5.78)	0.105
III	66	123.9 (119.9–127.9)	−1.66 (−6.93–3.60)	0.535	79.0 (76.0–82.0)	−1.34 (−5.32–2.63)	0.507
IV	44	127.0 (122.6–131.5)	1.48 (−4.37–7.32)	0.620	80.1 (76.7–83.4)	−0.30 (−4.71–4.11)	0.892
V	26	121.6 (115.6–127.7)	−3.94 (−11.09–3.21)	0.279	77.5 (72.9–82.1)	−2.88 (−8.27–2.52)	0.296
Continent							
Europe	125	123.6 (120.8–126.3)	Reference Category	Reference Category	79.9 (77.8–82.0)	Reference Category	Reference Category
Africa	44	131.9 (127.5–136.3)	8.35 (3.25–13.44)	0.001	87.7 (84.4–91.0)	7.79 (3.94–11.64)	<0.001
North America	234	126.3 (123.9–128.6)	2.69 (−1.07–6.44)	0.160	79.1 (77.3–80.8)	−0.83 (−3.66–2.01)	0.568
For SBP and DBP: Included participants *N* = 403; participants using antihypertensive medication or with missing information on use of antihypertensive medication were excluded (*N* = 41).							

	Prehypertension				Hypertension			
	*N*	Prevalence (95% CI)	OR (95% CI)	*P* value	*N*	Prevalence (95% CI)	OR (95% CI)	*P* value
Age								
18–29	212	21.4 (15.0–29.6)	Reference category	Reference category	225	21.5 (15.1–29.7)	Reference category	Reference category
30–39	107	19.2 (11.8–29.8)	0.88 (0.41–1.83)	0.729	117	30.8 (21.6–41.9)	1.63 (0.84–3.17)	0.151
≥40	84	27.2 (18.0–39.0)	1.38 (0.67–2.80)	0.380	100	44.3 (33.4–55.9)	2.91 (1.52–5.62)	0.001
Sex								
Women	192	18.3 (12.6–25.8)	Reference category	Reference category	215	27.6 (20.6–35.8)	Reference category	Reference category
Men	211	25.7 (19.5–33.2)	1.55 (0.88–2.76)	0.132	227	31.1 (24.2–38.9)	1.18 (0.71–1.97)	0.516
CP subtype								
Spastic	281	21.8 (17.1–27.5)	Reference category	Reference category	311	30.9 (25.4–37.0)	Reference category	Reference category
Other	37	24.1 (12.1–42.4)	1.14 (0.44–2.76)	0.780	40	19.8 (9.6–36.3)	0.55 (0.22–1.29)	0.186
CP distribution								
Unilateral	91	26.6 (15.2–42.2)	Reference category	Reference category	97	25.0 (14.0–40.6)	Reference category	Reference category
Bilateral	290	21.1 (16.0–27.2)	0.74 (0.32–1.70)	0.471	320	30.5 (24.6–37.1)	1.31 (0.59–3.03)	0.510
GMFCS								
I	128	20.1 (12.3–31.0)	Reference category	Reference category	138	27.2 (18.1–38.6)	Reference category	Reference category
II	129	18.3 (11.7–27.6)	0.89 (0.41–1.94)	0.775	139	39.4 (29.8–50.0)	1.75 (0.90–3.46)	0.105
III	66	36.4 (23.2–52.0)	2.27 (0.92–5.70)	0.076	73	19.3 (10.8–32.2)	0.64 (0.26–1.53)	0.323
IV	44	26.1 (13.8–43.8)	1.41 (0.48–4.07)	0.528	53	30.6 (17.9–47.1)	1.18 (0.46–3.02)	0.729
V	26	17.1 (6.2–39.3)	0.82 (0.19–2.96)	0.773	27	21.8 (8.9–44.5)	0.75 (0.20–2.43)	0.642
Continent								
Europe	125	24.0 (16.5–33.5)	Reference category	Reference category	130	17.9 (11.6–26.6)	Reference category	Reference category
Africa	44	24.2 (12.2–42.1)	1.01 (0.39–2.54)	0.981	53	46.0 (30.5–62.3)	3.90 (1.77–8.82)	<0.001
North America	234	20.3 (14.0–28.4)	0.81 (0.41–1.57)	0.524	259	33.5 (26.0–42.0)	2.31 (1.23–4.49)	0.011

Prehypertension is defined as SBP 130–139 mmHg and/or DBP 85–89 mmHg. Included participants *N* = 403; participants using antihypertensive medication or with missing information on antihypertensive medication were excluded (*N* = 41). Hypertension is defined as SBP at least 140 mmHg and/or DBP at least 90 mmHg or use of antihypertensive medication, following the European Hypertension guidelines (Williams, 2018). Included participants *N* = 442; participants with SBP less than 140 mmHg and DBP less than 90 mmHg and missing information on antihypertensive medication were excluded (*N* = 2). Linear and logistic multivariable regression models, including study as a fixed-effect were used to estimate the association of age, sex, cerebral palsy subtype, cerebral palsy distribution, GMFCS level and continent with the primary outcomes. Estimates were adjusted for these factors. Beta's (*β*) and odds ratios (ORs) and 95% CIs were estimated. A *P* value 0.05 or less was considered significant. CI, confidence interval; CP, cerebral palsy; GMFCS, Gross Motor Function Classification System; OR, odds ratio.

For SBP, BMI was a significant risk factor (*β* = 0.57, 95% CI 0.32–0.83, *P* < 0.001). For DBP, BMI (*β* = 0.38, 95% CI 0.18–0.58, *P* = 0.002) and resting heart rate (*β* = 0.22, 95% CI 0.10–0.33, *P* = 0.002) were significant risk factors. For hypertension, BMI (*β*= 1.10, 95% CI 1.05–1.15, *P* < 0.001) and alcohol consumption (*β* = 0.30, 95% CI 0.12–0.68, *P* = 0.038) were significant risk factors (Table 4).

**TABLE 4:** The effect of potential risk factors on SBP and DBP, prehypertension and hypertension following current European Hypertension guidelines

	SBP		DBP	
	Beta (95% CI)	*P* value	Beta (95% CI)	*P* value
Muscle tone (ref cat: no)	2.78 (−3.84–9.40)	0.816	0.94 (−4.46–6.35)	1.000
Family history of CVD (ref cat: no)	7.84 (0.83–14.84)	0.145	4.47 (−0.25–9.19)	0.316
BMI	0.57 (0.32–0.83)	<0.001	0.38 (0.18–0.58)	0.002
Resting heart rate	0.11 (−0.04–0.26)	0.543	0.22 (0.10–0.33)	0.002
Alcohol consumption (ref cat: no)	−5.69 (−10.15–−1.23)	0.089	−2.94 (−6.64–0.74)	0.467
Smoking (ref cat: no)	−0.96 (−7.15–5.24)	0.816	−0.04 (−4.86–4.78)	1.000

	Prehypertension		Hypertension	
	Beta (95% CI)	*P* value	Beta (95% CI)	*P* value
Muscle tone (ref cat: no)	0.51 (0.16–1.66)	1.000	2.29 (0.73–7.65)	0.650
Family history of CVD (ref cat: no)	4.55 (1.13–20.34)	0.295	0.78 (0.12–4.16)	1.000
BMI	1.03 (0.98–1.07)	1.000	1.10 (1.05–1.15)	<0.001
Resting heart rate	1.01 (0.99–1.04)	1.000	1.01 (0.99–1.04)	1.000
Alcohol consumption (ref cat: no)	1.55 (0.70–3.39)	1.000	0.30 (0.12–0.68)	0.038
Smoking (ref cat: no)	0.27 (0.06–0.89)	0.360	1.45 (0.52–3.98)	1.000

Data from Williams, 2018. Separate extended multivariable regression models were used, adjusted for one risk factor each. The models were adjusted for age, sex, CP subtype, CP distribution, GMFCS level and continent. *P* values were adjusted for multiple comparisons using the Holm method. CI, confidence interval; CP, cerebral palsy; CVD, cardiovascular disease; Ref cat, reference category.

Consistently, the European Hypertension guidelines for BP cutoffs were used; [12,33] however, we also examined the recently adapted American guidelines. Whenever using the American Hypertension guidelines, the overall prevalence of prehypertension was 11.8% (95% CI 7.6–16.9; Data Supplement S7) and the overall prevalence of hypertension was 61.4% (95% CI 51.0–71.3; Data Supplement S7). BP levels of more than half of the participants were classified as hypertension stage 1 (34%, SBP 130–139 mmHg or DBP 80–89 mmHg) or normal (30%, SBP <120 mmHg and DBP <80 mmHg) according to the American Hypertension guidelines (Data Supplement S7).

## DISCUSSION

This systematic review and meta-analysis combined BP data from six published and five unpublished studies and included 444 adults with cerebral palsy with a median age of 29.0 years, living on three different continents. The study indicated that the overall mean level of BP in adults with cerebral palsy was 124.9/79.9 mmHg and provides a reliable estimate of the overall prevalence of hypertension of 28.7%, according to current European Hypertension guidelines.

Our results suggest that in this young sample of adults with cerebral palsy, BP levels and the prevalence of hypertension are relatively high. Three reference studies were identified: a worldwide meta-analysis that established reference values for central BP and its amplification in a general healthy population (*n* = 45 436, mean age 49.6 years) [44]; a study that was performed by the Dutch National Institute for Public Health and the Environment (RIVM, ‘Nederland de maat genomen’, 2009–2010), measuring BP in the Dutch population according to age categories (*n* = 3865, mean age 52.3 years) [45] and a prospective cohort study in young adults (*n* = 4851, mean age 24.9 years at baseline) in the United States of America [46]. According to our findings, SBP was substantially higher in adults with cerebral palsy (124.9 mmHg, median age 29.0 years) than in American adults of a slightly younger age (110.4 mmHg, 24.9 years) [46] and comparable with two reference samples with a mean age of almost 20 years older (study 1: 126.2 mmHg, 49.6 years and 2: 126.1 mmHg, 52.3 years) [43,44]. DBP in adults with cerebral palsy (79.9 mmHg) was also higher than in all three reference studies (study 1: 75.4 mmHg, study 2: 77.3 mmHg, study 3: 68.6 mmHg). Prevalence of hypertension (28.7%) was higher in our study than in study 2 (23.9%) and 3 (13.2%). Importantly, BP was found to be comparable or higher in our relatively young adults with cerebral palsy. These comparisons should be interpreted with caution as only one reference study included international data, whereas the other two reference samples were national studies (Dutch and American). The prevalence of hypertension we found in our study was similar to a previous study in the USA in adults with cerebral palsy that described self-report data on hypertension from medical files. They found an incidence of hypertension of 30% in adults with cerebral palsy (*n* = 1015, mean age 58 years) compared with 22.1% in adults without cerebral palsy (*n* = 206 600, mean age 45 years) [17]. This study was limited by self-report data, which might be susceptible to response bias, and their mean age was higher compared with our sample; however, these findings also suggest that adults with cerebral palsy are at risk for hypertension.

Subgroup analyses indicated that higher BP levels and prevalence of hypertension were found in adults with cerebral palsy above 40 years of age or those who lived in Africa. In addition, SBP levels were higher among men or those with spastic cerebral palsy. Age-related changes in BP are consistent with findings in the general population, where hypertension becomes progressively more common with advancing age [47]. This could be related to the large increases in arterial stiffness, which seems to progress faster and at a younger age in adults with cerebral palsy compared with the general population [48]. SBP levels were higher in men than in women, which is consistent with findings in the general population [49].

To date, little attention has been given to subgroups of adults with cerebral palsy regarding the risk for hypertension. Higher BP levels were expected in spastic cerebral palsy, based on clinical experience, and previous studies in stroke patients [50,51]. In stroke patients, BP was found to be significantly higher in paretic arms of patients with a spastic tone and lower in arms with a flaccid tone. Accordingly, measuring BP in the unaffected arm was recommended. Our results suggest that SBP is higher in adults with spastic cerebral palsy than in adults with other subtypes of cerebral palsy. This suggests that spasticity might affect BP levels in adults with cerebral palsy. Little is known regarding the precise effect of spasticity on blood vessels or on the BP measurement itself in either cerebral palsy or other diagnoses with spasticity (i.e. whether the higher BP measured is representative of an increased CVD risk or rather a mechanical effect because of the increased muscle tone). In our meta-analysis, almost half of the included studies measured BP in the least affected or unaffected arm, whereas others used the left or right arm not taking into account whether this arm was affected by spasticity. It is important to acknowledge that 70% of participants had a bilateral distribution of cerebral palsy, so elevated tone might have been present in the least affected arm as well. It should also be noted that the majority of adults with cerebral palsy in this study had spastic cerebral palsy; only 10% had other subtypes of cerebral palsy, which consisted of dyskinetic or ataxic cerebral palsy, often in combination with spastic cerebral palsy. As spasticity is the most common motor abnormality in persons with cerebral palsy and affected arms are often underdeveloped in cerebral palsy, future research should investigate the influence of tone and/or contractures and its underlying mechanisms on BP levels in adults with cerebral palsy. In fact, we suggest that central BP measurement should be used as an accurate measure of BP.

Higher BP levels were also expected in more severely affected adults with cerebral palsy (e.g. bilateral distribution and lower levels of gross motor functioning) as a consequence of a more sedentary and less active lifestyle [52]. Surprisingly, analyses of cerebral palsy distribution and GMFCS levels revealed no differences in BP levels. An explanation for this result on cerebral palsy distribution is that adults with cerebral palsy with bilateral distribution can be diplegic or tetraplegic. Therefore, it is possible that some of those adults maintain mobility and manage to be active. Although unexpected, this finding might reflect bias towards a healthier segment of the cerebral palsy population with GMFCS IV and V. Indeed many factors can contribute to hypertension, including excess visceral adiposity [53], which was found to be present in adults with cerebral palsy [54]. We were limited in our ability to explore all factors that are associated with elevated BP, and thus future research is needed to examine additional mechanisms that explain why some subtypes of cerebral palsy are at higher risk for hypertension.

Another key finding in the subgroup analysis was that BP levels and prevalence of hypertension were highest in adults with cerebral palsy who lived in Africa, only DBP levels were higher in North America. The external validity of these findings should be interpreted cautiously as a variety of factors could have influenced BP levels. For some of the participants in Africa, it was the first time their BP was measured, so heightened sympathetic nervous system activity (e.g. anxiety) could be an unaccounted factor. Additionally, adults with cerebral palsy do not have regular BP assessments, partly because of travel time to the clinic. Finally, although some participants in these cohorts from Africa used antihypertensive medication, for others, these medications might be unaffordable.

BMI, resting heart rate and alcohol consumption were factors that influenced SBP or DBP levels or hypertension. No significant results were found for muscle tone, family history of CVD and smoking. These data must be interpreted with caution because of missing data, large confidence intervals, and our limited ability to include all covariates in a single model. More research is needed to investigate the exact effect of potential risk factors on BP in adults with cerebral palsy. Some of these factors are modifiable, which emphasizes the importance of stimulating a healthy lifestyle with more physical activity and a healthy diet and might be impetus for a behavioral intervention to regulate BP in adults with cerebral palsy.

Early detection of hypertension in the general population can prevent end-organ damage, such as CVD. As a higher risk of CVD was seen in adults with cerebral palsy than in the general population [13], it is of importance to focus on modifiable risk factors, such as BP. BP is one of the eight outcomes included in the final Core Outcome Set of Measurement Instruments for assessing multimorbidity risk in adults with cerebral palsy [55]. We, therefore, recommend that regular clinical checks and monitoring of BP should be included in their standard care. In some of the countries included in this study, that is, the United States of America and Finland, BP measurements are included in standard health clinic procedures, whereas in other countries, it is not. More attention should be given to diagnosing hypertension in adults with cerebral palsy, which should start at a young adult age. Further research should focus on whether prevention, treatment and management of hypertension in adults with cerebral palsy could be similar to the general population.

As shown in our study, prevalence estimates of hypertension depend on the cut-off used to define hypertension. In 2017, the definition of hypertension in the American Hypertension guidelines was changed from at least 140/90 mmHg to at least 130/80 mmHg. This results in a higher prevalence of hypertension, mainly as many adults with cerebral palsy in our meta-analysis had BP values between these cut-off values; 137 (34.0%) adults with cerebral palsy had BP values in hypertension stage 1 (SBP 130–139 mmHg or DBP 80-89 mmHg). In general, lowering the cut point might result in more awareness and prevention but it would also increase the number of people being prescribed antihypertensive medication, which increases the costs of health care.

The strength of this meta-analysis is that samples from published and unpublished studies measuring BP in adults with cerebral palsy were included, resulting in a large sample of 444 adults with cerebral palsy covering all GMFCS levels and a wide age range. There are also limitations to this study. A factor that may have affected the results is the method of BP measurements. Most of the studies included in our meta-analysis followed the guideline for measuring BP [33]. Nonetheless, different BP devices were used, and almost half of the studies measured it only once, whereas at least two measurements are recommended. As all data were collected for research purposes, BP measurements were all taken on the same day. In case of hypertensive levels in clinical practice, BP is re-measured after 2 weeks, which will minimize white-coat syndrome, random error and provide a more accurate basis for estimation of BP. Therefore, some caution is needed when interpreting our results. As different measurement methods likely influence BP levels, consensus is needed to standardize the method of measurement of BP in adults with cerebral palsy. For the above-mentioned Core Outcome Set of Outcome Measurement Instruments, it was recommended to measure BP with a calibrated device and appropriate sized cuff, in a seated position, after 10 min of rest, on the least affected side [55]. The most recent guideline recommends repeated unsupervised measurements as used in the hallmark study SPRINT [56]. Further research could fine-tune the optimal method to measure BP in adults with cerebral palsy, taking into account the potential effect of spasticity, as mentioned earlier in the discussion. It would be interesting to correlate office measurements to the gold standard, 24-h ambulatory BP measurement, to also allow conclusions about white-coat effect and masked hypertension in adults with cerebral palsy.

Another limitation is that our sample was relatively young. In order to draw conclusions across the lifespan of cerebral palsy, it is recommended to include older adults with cerebral palsy in future research. In addition, results on subgroup analyses should be interpreted with caution because of few observations in some subgroups (e.g. GMFCS level V); however, these distributions correspond with the general cerebral palsy population. Additionally, studies were performed in three different parts of the world, including North America, Europe and Africa, but not all World Health regions were represented, which limits generalizability beyond these populations. Another limitation is that secondary outcomes were assessed by a variety of scales and required conversion to a common scale (e.g. muscle tone was measured by different scales and in different muscles), which was not always possible. To facilitate comparison across studies, health care institutions and countries, we need to make sure that outcome assessment is standardized. The Core Outcome Set of Outcome Measurement Instruments for assessing multimorbidity risk in adults with cerebral palsy [55] is a good example but consensus is also needed for other outcomes, for example, pain and fatigue. The implementation of the ICF Core Set for adults with cerebral palsy, which is currently under development, could also contribute to this [57]. Finally, it should be noted that some samples may be biased. Participants from individual studies were recruited through flyers and advertisements, patient registry databases and rehabilitation clinics, assuming the use of convenience samples. However, patients looking after their health are more willing to respond to advertisements or calls for research, which might have resulted in an underestimation of the true BP levels in adults with cerebral palsy.

The results of this meta-analysis in a relatively young cohort indicate that almost 30% of adults with cerebral palsy are hypertensive. We, therefore, recommend clinical screening for BP in adults with cerebral palsy beginning in young adulthood.

## ACKNOWLEDGEMENTS

We thank Wichor Bramer, information specialist, Medical Library, Erasmus MC University Medical Center Rotterdam, Rotterdam, the Netherlands, for his help with the literature search strategy. We thank everyone involved in one of the included studies for their contributions, specifically: Dr James J. Carollo, Center for Gait and Motion Analysis, Children's Hospital Colorado, Aurora, Colorado, USA, Dr Deborah Gaebler-Spira, Department of Physical Medicine and Rehabilitation, Northwestern University Feinberg School of Medicine, Chicago, Illinois, USA, Shirley Ryan AbilityLab, Chicago, Illinois, USA, Dr Saroj Saigal, McMaster University, Hamilton and Professor Dr Jan Willem Gorter, Department of Pediatrics, McMaster University, Institute for Applied Health Sciences, Hamilton, Canada, and Dr Jorrit Slaman, Rijndam Rehabilitation, Rotterdam, the Netherlands.

### Conflicts of interest

This study was funded by Rijndam Rehabilitation, Rotterdam, The Netherlands. Dr. Thorpe's work was funded in part by an NIH Career Award (#5K23RR24054) and by the Cerebral Palsy International Research Foundation, Ethel Hausman Clinical Scholars Award. Dr. Heyn was supported by grants from the National Institute on Disability, Independent Living, and Rehabilitation Research (NIDILRR #H133G130200, NIDILRR #90IF0055-01), in the Administration for Community Living (ACL) of the Department of Health and Human Services (HHS). Additional support was provided from the J.T. Tai & Company Foundation. Prof. Marciniak's work was funded by the Shirley Ryan AbilityLab. Funding of Dr. Morrison's cohort was provided by the Canadian Institutes of Health Research. For the remaining authors, no conflicts of interest or sources of funding were declared.

## Supplementary Material

Supplemental Digital Content
