# Supplementary material for: Blood pressure in adults with cerebral palsy: a systematic review and meta-analysis of individual participant data
Source: J Hypertens. 2021 Jun 7;39(10):1942–55. doi: 10.1097/HJH.0000000000002912 (PMC8452335; doi:10.1097/HJH.0000000000002912)
Supplement: Supplemental Digital Content [file jhype-39-1942-s001.docx]

SUPPLEMENTAL DIGITAL CONTENT

- S1. Search strategy
- S2. Method of conversion to common scales or outcome measures
- S3. Personal characteristics and mean levels of CP-related factors, biological and lifestyle-related risk factors of the total sample
- S4. Scores on the methodological quality assessment of included studies
- S5. Information on blood pressure measurements
- S6. Funnel plots for systolic and diastolic blood pressure
- S7. Forest plots for overall prevalence of prehypertension and hypertension, and classification of blood pressure following current American Hypertension guidelines (Whelton, 2018)

**S1.** Search strategy

**Embase.com**

('blood pressure'/exp OR 'blood pressure monitoring'/exp OR 'abnormal blood pressure'/exp OR 'blood pressure measurement'/de OR 'hypertension encephalopathy'/de OR 'blood pressure monitor'/exp OR (((blood OR vessel* OR vascul* OR intravascul* OR venous OR arter*) NEAR/3 (pressure OR tension*)) OR hypotens* OR hypertens* OR prehypertens* OR normotens* OR ankle-brachial-ind*):ab,ti) AND ('cerebral palsy'/exp OR 'spastic paresis'/de OR (((cerebral* OR brain OR spastic ) NEXT/3 (pals* OR paralys* OR hemipleg* OR diplegi* OR paresis)) OR 'encephalopathia infantilis' ):ab,ti) NOT ([animals]/lim NOT [humans]/lim) NOT (child/exp NOT adult/exp)

**Medline Ovid**

(exp "blood pressure"/ OR exp "Hypertension"/ OR Prehypertension/ OR exp Hypotension/ OR exp "Blood Pressure Determination"/ OR "Blood Pressure Monitors"/ OR (((blood OR vessel* OR vascul* OR intravascul* OR venous OR arter*) ADJ3 (pressure OR tension*)) OR hypotens* OR hypertens* OR prehypertens* OR normotens* OR ankle-brachial-ind*).ab,ti,kf.) AND ("cerebral palsy"/ OR (((cerebral* OR brain OR spastic ) ADJ3 (pals* OR paralys* OR hemipleg* OR diplegi* OR paresis)) OR "encephalopathia infantilis" ).ab,ti,kf.) NOT (exp animals/ NOT humans/) NOT ((exp child/ OR exp infant/) NOT exp adult/)

**PsycINFO Ovid**

(exp "blood pressure"/ OR exp "Hypertension"/ OR exp Hypotension/ OR (((blood OR vessel* OR vascul* OR intravascul* OR venous OR arter*) ADJ3 (pressure OR tension*)) OR hypotens* OR hypertens* OR prehypertens* OR normotens* OR ankle-brachial-ind*).ab,ti.) AND ("cerebral palsy"/ OR (((cerebral* OR brain OR spastic) ADJ3 (pals* OR paralys* OR hemipleg* OR diplegi* OR paresis)) OR "encephalopathia infantilis" ).ab,ti.) NOT (exp animals/ NOT humans/) NOT ((100.ag.) NOT 300.ag.)

**CINAHL EBSCOhost**

(MH "blood pressure+" OR MH "Blood Pressure Devices+" OR MH "Blood Pressure Determination+" OR MH "Hypertension+" OR MH Hypotension+ OR TI (((blood OR vessel* OR vascul* OR intravascul* OR venous OR arter*) N2 (pressure OR tension*)) OR hypotens* OR hypertens* OR prehypertens* OR normotens* OR ankle-brachial-ind*) OR AB (((blood OR vessel* OR vascul* OR intravascul* OR venous OR arter*) N2 (pressure OR tension*)) OR hypotens* OR hypertens* OR prehypertens* OR normotens* OR ankle-brachial-ind*)) AND (MH "cerebral palsy" OR TI (((cerebral* OR brain OR spastic ) N2 (pals* OR paralys* OR hemipleg* OR diplegi* OR paresis)) OR "encephalopathia infantilis" ) OR AB (((cerebral* OR brain OR spastic ) N2 (pals* OR paralys* OR hemipleg* OR diplegi* OR paresis)) OR "encephalopathia infantilis" )) NOT (MH animals+ NOT MH humans+) NOT ((MH child+ OR MH infant+) NOT MH adult+)

**Cochrane CENTRAL**

((((blood OR vessel* OR vascul* OR intravascul* OR venous OR arter*) NEAR/3 (pressure OR tension*)) OR hypotens* OR hypertens* OR prehypertens* OR normotens* OR ankle-brachial-ind*):ab,ti) AND ((((cerebral* OR brain OR spastic ) NEXT/3 (pals* OR paralys* OR hemipleg* OR diplegi* OR paresis)) OR 'encephalopathia infantilis' ):ab,ti) NOT (([mh ^child] OR [mh ^infant]) NOT [mh ^adult])

**Web of Science**

TS=(((((blood OR vessel* OR vascul* OR intravascul* OR venous OR arter*) NEAR/2 (pressure OR tension*)) OR hypotens* OR hypertens* OR prehypertens* OR normotens* OR ankle-brachial-ind*)) AND ((((cerebral* OR brain OR spastic ) NEAR/2 (pals* OR paralys* OR hemipleg* OR diplegi* OR paresis)) OR "encephalopathia infantilis" )) NOT ((child* OR infant* OR neonat* OR newborn*) NOT adult*))

**Google Scholar**

"blood|intravascular|venous|arterial pressure| tension"| hypotension| hypertension| prehypertension| normotension|"ankle-brachial-index" "cerebral|brain| spastic palsy| paralysis| hemiplegia| diplegia| paresis"| "encephalopathia infantilis"

**S2.** Methods of conversion to common scales or outcome measures

| **Outcome and measurement instrument** | **Question or item and answer or scores** | **Method of conversion** |
| --- | --- | --- |
| **Intellectual disability** | Defined as a moderate to severe level of intellectual functioning, indicated as an IQ level below 70 (DSM-5) | ***Yes, no*** |
| Short Test of Mental Status (STMS)^[1]^  Heyn et al^[2]^ | Short Test of Mental Status total score  A. Orientation Score, B. Attention, C. Learning, D. Calculation, E. Abstraction, F. Information, G. Construction, H. Recall | *Total score 1-28=yes*  *Total score 29-38=no* |
| Mental retardation  Thorpe et al^[3]^ | *Yes, no* | *Idem* |
| IQ  Morrison et al^[4]^ |  | *IQ ≤ 70=yes*  *IQ > 70=no* |
| Cognitive functioning  Salokivi et al^[5]^ | Moderate cognitive functioning: at the level of 6-8 years of age, Severe cognitive functioning: at the level of 3-5 years of age, Very severe cognitive functioning: at the level of 2 years of age or under | *All yes* |
| Exclusion criteria severe cognitive impairments  van den Berg-Emons et al^[6]^  van der Slot et al^[7]^ |  | *All no* |
| No severe communication or understanding problems that impede proper measurement performance  Verschuren et al, unpublished data, 2015-2016 |  | *All no* |
| Not included in study  Lamberts et al, unpublished data, 2017  Langerak et al, unpublished data, 2017 |  | *All no* |
| **Muscle tone** | Tone in hip adductors, knee extensors, knee flexors, ankle plantar flexors, elbow flexors, wrist flexors  Normal tone: No increase in muscle tone  Hypertonia: Slight increase in muscle tone, manifested by a catch and release or by minimal resistance throughout the remained (less than half) of the range of motion or at the end when the affected part(s) is moved in flexion or extension, more marked increase in muscle tone through most of the range of motion, but affected part(s) easily flexed, considerable increase in tone, passive movement difficult, affected part(s) rigid in flexion or extension  If one of the muscles is hypertone, than hypertonia; otherwise normal tone | ***Normotonic, hypertonic*** |
| Modified Ashworth Scale^[8]^  Heyn et al^[2]^  Marciniak et al^[9]^  van den Berg-Emons et al^[6]^ | Muscles: knee extensors, knee flexors, ankle plantar flexors, left and right  *no increase in muscle tone (0), slight increase in muscle tone, manifested by a catch and release or by minimal resistance at the end of the range of motion when the affected part(s) is moved in flexion or extension (1), slight increase in muscle tone, manifested by a catch and release or by minimal resistance throughout the remainder (less than half) of the range of motion (1+), more marked increase in muscle tone through most of the range of motion, but affected part(s) easily flexed (2), considerable increase in tone, passive movement difficult affected part(s) (3), rigid in flexion or extension (4)*  Heyn et al^[2]^  Muscles: knee extensors, knee flexors, elbow flexors, wrist flexors, left and right  *no increased tone (0), catch but no increased tone through the remainder of the range or catch with increased tone through < 50 percent of the range (1), catch followed by increased tone through greater than 50 percent of the range of the joint (2), moderate to marked increased tone throughout (3), rigid in flexion or extension (4)*  Marciniak et al^[9]^  Muscles: hip adductors, knee extensors, knee flexors, ankle plantar flexors elbow flexors, wrist flexors, left and right  *no increase in muscle tone (0), slight increase in muscle tone, manifested by a catch and release or by minimal resistance at the end of the range of motion when the affected part(s) is moved in flexion or extension (1), slight increase in muscle tone, manifested by a catch and release or by minimal resistance throughout the remainder (less than half) of the range of motion (1+), more marked increase in muscle tone throughout most of the range of motion (2), considerable increase in tone, passive movement difficult (3), rigid in flexion or extension (4)*  van den Berg-Emons et al^[6]^ | *0= normal tone*  *1-4= hypertonia* |
| Ashworth Scale^[10]^  Lamberts et al, unpublished data, 2017  Langerak et al, unpublished data, 2017 | Muscles: hip adductors, knee extensors, knee flexors, ankle plantar flexors, left and right  *no increase in tone (0), slight increase in tone giving a catch when limb is moved in flexion/extension (1), more marked increase in tone but limb easily flexed (2), considerable increase in tone, passive movement difficult (3), limb rigid in flexion or extension (4)*  Lamberts et al, unpublished data, 2017  Langerak et al, unpublished data, 2017 | *0= normal tone*  *1-4= hypertonia* |
| Clinical tone measurement  van der Slot et al^[7]^ | Arm, leg, left and right  *Normal, hypertone, spastic* | *Normal= idem, hypertone=idem, spastic=hypertonia* |
| **Pain** |  | ***None, very mild, mild, moderate, severe, very severe*** |
| Patient-Reported Outcomes Measurement Information System (PROMIS) - 57 Profile (v2.1)^[11]^  Heyn et al^[2]^ | Item 57. In the past 7 days, how would you rate your pain on average?  *No pain (0) - worst imaginable pain (10)* | *No pain (0)=none, 1,2=very mild, 3,4=mild, 5,6=moderate, 7,8=severe, 9, 10=very severe* |
| Wong-Baker FACES Pain Rating Scale^[12]^  Thorpe et al^[3]^ | Ask the person to choose the face that best describes how he/she is feeling  *No pain, hurts a little, hurts a little more, hurts even more, hurts a whole lot, hurts as much as you can imagine* | *No pain=none, hurts a little=very mild, hurts a little more=mild, hurts even more=moderate, hurts a whole lot=severe, hurts as much as you can imagine=very severe* |
| Short Form Health Survey (SF-36)^[13]^  van den Berg-Emons et al^[6]^  van der Slot et al^[7]^  Lamberts et al, unpublished data, 2017  Langerak et al, unpublished data, 2017 | Item 7. Bodily pain: How much bodily pain have you had during the past 4 weeks?  *None, very mild, mild, moderate, severe, very severe* | *Idem* |
| **Fatigue** |  | ***No fatigue, fatigue, severe fatigue*** |
| Patient-Reported Outcomes Measurement Information System (PROMIS) - 57 Profile v2.1^[11]^  Heyn et al^[2]^ | Item 25. I feel fatigued  Item 26. I have trouble starting things because I am tired  Item 27. How run-down did you feel on average?  Item 28. How fatigued were you on average?  Item 29. How much were you bothered by your fatigue on average?  Item 30. To what degree did your fatigue interfere with your physical functioning?  Item 31. How often did you have to push yourself to get things done because of your fatigue?  Item 32. How often did you have trouble finishing things because of your fatigue?  *Not at all (1), a little bit (2), somewhat (3), quite a bit (4), very much/always (5)* | T-score < 55 within normal limits = no fatigue, T-score 55-60 mild= fatigue, T-score 60-70 moderate=fatigue, T-score > 70 severe=severe fatigue |
| Fatigue Severity Scale (FSS)^[14]^  van den Berg-Emons et al^[6]^  van der Slot et al^[7]^  Lamberts et al, unpublished data, 2017  Langerak et al, unpublished data, 2017 | During the past week, I have found that:  Item 1. My motivation is lower when I am fatigued.  Item 2. Exercise brings on my fatigue.  Item 3. I am easily fatigued.  Item 4. Fatigue interferes with my physical functioning.  Item 5. Fatigue causes frequent problems for me.  Item 6. My fatigue prevents sustained physical functioning.  Item 7. Fatigue interferes with carrying out certain duties and responsibilities.  Item 8. Fatigue is among my three most disabling symptoms.  Item 9. Fatigue interferes with my work, family, or social life.  *Strongly disagree with the statement (1) - strongly agree with the statement. (7)* | FSS < 4.0=no fatigue, FSS 4.0 - 5.0= fatigue, FSS ≥ 5.1=severe fatigue |
| **Aerobic fitness** | Patients included in case of maximum exertion, based on Respiratory Exchange Ratio (RER) for different sex and age groups (Edvardsen, 2014)  Male and female  20-49y RER ≥ 1.10  50-64y RER ≥ 1.05  ≥65y RER ≥ 1.00 | ***VO2 max (ml/kg/min)*** |
| Progressive ramp protocol on electronically braked cycle ergometers (Jaeger ER800; Jaeger Tonnies, Breda, The Netherlands or Corival V2 Lode B.V., Groningen, The Netherlands)  van den Berg-Emons et al^[6]^ |  | *Idem* |
| Electronically braked cycle ergometer according to the McMaster All-Out Progressive Continuous Protocol (Jaeger ER 800; Jaeger Tonnies, Breda, The Netherlands)  van der Slot et al^[7]^ |  | *Idem* |
| 10m Shuttle walk/run test  Lamberts et al, unpublished data, 2017  Langerak et al, unpublished data, 2017 |  | *Idem* |
| **Physical activity** |  | ***In %*** |
| Ambulatory Monitoring system VitaMove (2M Engineering, Veldhoven, The Netherlands)  van den Berg-Emons et al^[6]^ | Physical activity including standing, general movement, walking, wheelchair driving, cycling, running | *Idem* |
| Accelerometry-based Activity Monitor (AM; TEMEC Instruments, Kerkrade, The Netherlands)  van der Slot et al^[7]^ | Physical activity including standing, standing public transport, general movement, walking, wheelchair driving, cycling, running, climbing up, climbing down | *Idem* |

**S3.** Personal characteristics and mean levels of CP-related factors, biological and lifestyle-related risk factors of the total sample (N=444).

| **Personal characteristics** | |
| --- | --- |
| Intellectual disability n (%) | N=353; Md=91 |
| No | 300 (85) |
| Yes | 53 (15) |
| Education n (%) | N=287; Md=157 |
| Prevocational practical education or less | 81 (28) |
| Prevocational theoretical education and secondary education | 65 (23) |
| Higher vocational education and university | 119 (41) |
| Other | 22 (8) |
| Employment n (%) | N=218; Md=226 |
| School / study | 48 (22) |
| Paid job | 102 (47) |
| Volunteer work | 14 (7) |
| Day-center occupation | 3 (1) |
| Unemployed / seeking for jobs | 33 (15) |
| Occupationally disabled | 18 (8) |
| Civil status n (%) | N=319; Md=125 |
| Single | 257 (81) |
| Partner / married | 58 (18) |
| Separated / widow | 4 (1) |
| Living situation n (%) | N=198; Md=246 |
| Alone | 94 (47) |
| With partner (and children) | 14 (7) |
| With parents / family | 61 (31) |
| With others, group home | 29 (15) |
| Continent n (%) | N=444; Md=0 |
| Europe | 132 (30) |
| Africa | 53 (12) |
| North America | 259 (58) |
| **Cerebral palsy-related factors** | |
| Muscle tone n (%) | N=260; Md=184 |
| Normal tone | 27 (10) |
| Hypertonia | 233 (90) |
| Pain n (%) | N=304; Md=140 |
| None | 135 (44) |
| Very mild | 58 (19) |
| Mild | 64 (21) |
| Moderate | 33 (11) |
| Severe | 12 (4) |
| Very severe | 2 (1) |
| Fatigue n (%) | N=218; Md=226 |
| No fatigue | 133 (61) |
| Fatigue | 54 (25) |
| Severe fatigue | 31 (14) |
| **Biological risk factors** | |
| Family history of CVD n (%) | N=155; Md=289 |
| No | 85 (55) |
| Yes | 70 (45) |
| BMI (kg/m^2^) | N=397; Md=47 |
|  | 25.10 ± 6.14 |
| Waist-to-hip ratio (in stance) | N=132; Md=312 |
|  | 0.94 ± 0.14 |
| Resting heart rate (beats / min) | N=251; Md=193 |
|  | 80.32 ± 14.77 |
| Aerobic fitness - VO2max (ml/kg/min) | N=101; Md=343 |
|  | 33.03 ± 9.31 |
| Total cholesterol (mmol/L) | N=194; Md=250 |
|  | 4.59 ± 0.94 |
| HDL (mmol/L) | N=194; Md=250 |
|  | 1.37 ± 0.42 |
| LDL (mmol/L) | N=193; Md=251 |
|  | 2.78 ± 0.83 |
| TC/HDL ratio | N=194; Md=250 |
|  | 3.66 ± 1.55 |
| Triglycerides (mmol/L) | N=192; Md=252 |
|  | 1.15 ± 0.72 |
| Glucose (mmol/L) | N=173; Md=271 |
|  | 4.93 ± 0.80 |
| Insulin (IU) | N=115; Md=329 |
|  | 10.25 ± 8.57 |
| Diabetes n (%) | N=294; Md=150 |
| No | 290 (99) |
| Yes | 4 (1) |
| **Lifestyle-related risk factors** | |
| Alcohol consumption n (%) | N=329; Md=115 |
| No | 240 (73) |
| Yes | 89 (27) |
| Smoking n (%) | N=307; Md=137 |
| No | 269 (88) |
| Yes | 38 (12) |
| Physical activity (%) | N=89; Md=355 |
|  | 18.90 ± 8.03 |

Md= missing data; BMI= body mass index; CVD= cardiovascular disease

**S4**. Scores on the methodological quality assessment of included studies

| **Study** | **Title/ abstract** | **Methods** | | | | | | **Results** | | **Discussion** | | **Total score** |
| --- | --- | --- | --- | --- | --- | --- | --- | --- | --- | --- | --- | --- |
|  | **1. Title and abstract** | **2. Study design** | **3. Setting** | **4. Participants** | **5. Variables** | **6. Data sources/ measurement** | **7. Bias** | **8. Participants** | **9. Descriptive data** | **10. Limitations** | **11. Generalizability** |  |
| Heyn et al^[2]^ | 1 | 1 | **1** | 1 | **1** | 1 | 1 | 1 | **1** | 1 | 1 | 11 |
| Van der Slot et al^[7]^ | 1 | 1 | **1** | 1 | 1 | 1 | 1 | 1 | 1 | 1 | 1 | 11 |
| Van den Berg-Emons et al^[6]^ | 1 | 1 | **1** | 1 | **1** | 1 | **1** | 1 | 0.5 | 1 | 1 | 10.5 |
| Morrison et al^[4]^ | 1 | 1 | 1 | 1 | 1 | 1 | **1** | 1 | 1 | 0.5 | 1 | 10.5 |
| Marciniak et al^[9]^ | 1 | 1 | **1** | **1** | 1 | **1** | 0 | **1** | **1** | **1** | **0.5** | 9.5 |
| Lamberts et al, unpublished data, 2017 | **1** | **1** | **1** | **1** | **0.5** | **1** | **1** | **1** | **1** | **0.5** | **0** | 9 |
| Langerak et al, unpublished data, 2017 | **1** | **1** | **1** | **1** | **0.5** | **1** | **1** | **1** | **1** | **0.5** | **0** | 9 |
| McPhee et al^[15]^ | 1 | 1 | 0.5 | 0.5 | 1 | 1 | 0 | 1 | 1 | 1 | 1 | 9 |
| Thorpe et al^[3]^ | 1 | 1 | **1** | **1** | 0.5 | 0.5 | 0 | **1** | 0.5 | **1** | 1 | 8.5 |
| Verschuren et al, unpublished data, 2015-2016 | **0** | **1** | **1** | **1** | **1** | **1** | **0.5** | **1** | **1** | **0.5** | **0.5** | 8.5 |
| Salokivi et al^[5]^ | **1** | **1** | **1** | **1** | 0.5 | 0 | 0 | 0.5 | 0 | 0 | 0 | 5 |

Rating: 1=yes, 0.5=partially, 0=no. Bold numbers indicate that these ratings are based upon additional information derived from other publications of the same study sample or clarification from the primary investigator.

Selected items from Strengthening the Reporting of Observational Studies in Epidemiology (STROBE) Statement (von Elm, 2014). 1. Title and abstract: Is the study’s design indicated with a commonly used term in the title or the abstract? Is an informative and balanced summary of what was done and what was found provided in the abstract? 2. Study design: Are key elements of study design presented early in the paper? 3. Setting: Are settings, locations, and relevant dates, including periods of recruitment, exposure, follow-up and data collection described? 4. Participants: Are the eligibility criteria, and the sources and methods of selection of participants given? 5. Variables: Are all outcomes, exposures, predictors, potential confounders, and effect modifiers clearly defined? Are diagnostic criteria given (if applicable)? 6. Data sources/ measurement: For each variable of interest, are sources of data and details of methods of assessment (measurement) given? 7. Bias: Are any efforts to address potential sources of bias described? 8. Participants: Are the numbers of individuals at each stage of the study reported– e.g., numbers potentially eligible, examined for eligibility, confirmed eligible, included in the study, completing follow-up, and analyzed. Are reasons for non-participation at each stage described? 9. Descriptive data: Are characteristics of study participants (e.g., demographic, clinical, social) and information on exposures and potential confounders given? Is number of participants with missing data for each variable of interest indicated? 10. Limitations: Are limitations of the study, taking into account sources of potential bias or imprecision, discussed? Are both direction and magnitude of any potential bias discussed? 11. Generalizability: Is the generalizability (external validity) of the study results discussed?

**S5.** Information on blood pressure measurements

| **Samples** | **Device:**  **type (brand)** | **Number of BP measure-ments per participant** | **Properly maintained, calibrated, and validated device and appropriately sized cuff** | **Patient selection (hyperten-sive patients selected)** | **Side of measure-ments** | **Cuff positioned at level of heart** | **Position** | **Five minutes rest before measurement** | **At least 30 minutes prior to the measurement:** | | |
| --- | --- | --- | --- | --- | --- | --- | --- | --- | --- | --- | --- |
|  |  |  |  |  |  |  |  |  | **No exercise** | **No smoking and no caffeine** | **No alcohol** |
| Heyn et al^[2]^ | Digital (WelchAllyn) | 1 | Yes | No | Right arm | Yes | Seated | Yes | Yes | Yes | Yes |
| Marciniak et al^[9]^ | Digital (Dinamap) | Average of 3 | Yes | No | Unaffected/ least affected side | Yes | Seated | Yes | Yes | Yes | Yes |
| Thorpe et al^[3]^ | Digital  (GE Procare 400) | 1 | Yes | No | Dominant or unaffected/ least affected side | Yes | Seated | Yes | Yes | Yes | Yes |
| McPhee et al^[15]^ | Digital  (Dinamap Pro 100, Critikon LCC, Tampa, Fla, USA) | 4 | Yes | No | Right arm | Yes | Supine | Yes | Yes | Yes | Yes |
| Morrison et al^[4]^ | Oscillometic  (BP Tru, BPM 300, VSM Medtech Ltd, Vancouver, BC) | 5 | Yes | No | Unaffected/ least affected side | Yes | Seated | Yes | Yes | Yes | Yes |
| van den Berg-Emons et al^[6]^ | Manual  (Speidel and Keller Maxi Stabil 3) | 2 | Yes | No | Left arm | Yes | Seated | Yes | Yes | Yes | Yes |
| van der Slot et al^[7]^ | Manual  (Maxi Stabil, Speidel & Keller, Germany) | 2 | Yes | No | Unaffected/ least affected side | Yes | Seated | Yes | Yes | Yes | Yes |
| Verschuren et al, unpublished data, 2015-2016 | Digital  (Omron Healthcare, Model M3, Kyoto) | 1 | Yes | No | Unaffected/ least affected side | Yes | Seated | Yes | Yes | Yes | Yes |
| Salokivi et al^[5]^ | Digital  (Omron) | 1 | Yes | No | Left arm | Yes | Both | Yes | Yes | Yes | Yes |
| Lamberts et al, unpublished data, 2017 | Digital  (Omron MIT Elite Plus) | 2 | Yes | No | Left arm | Yes | Seated | Yes | Yes | Yes | Yes |
| Langerak et al, unpublished data, 2017 | Digital  (Omron MIT Elite Plus) | 2 | Yes | No | Left arm | Yes | Seated | Yes | Yes | Yes | Yes |

**S6**. Funnel plots for systolic and diastolic blood pressure

Funnel plot for systolic blood pressure


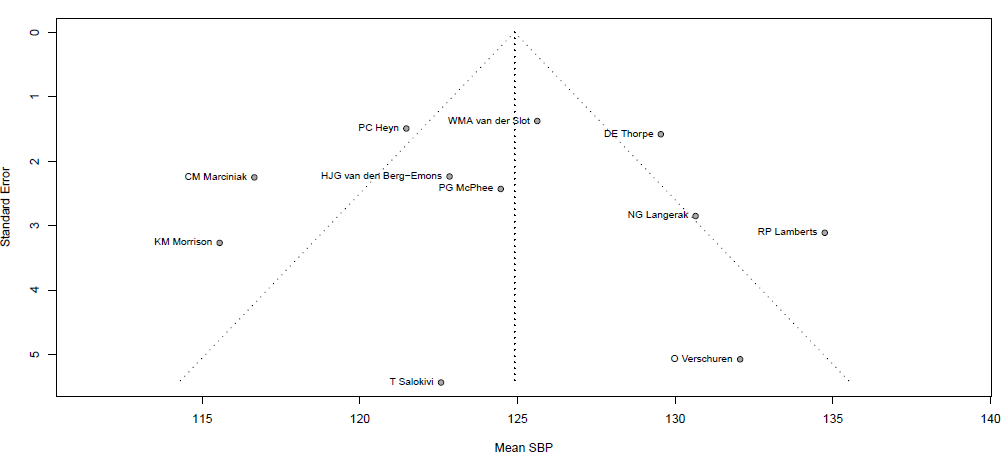


SBP=systolic blood pressure

Funnel plot for diastolic blood pressure


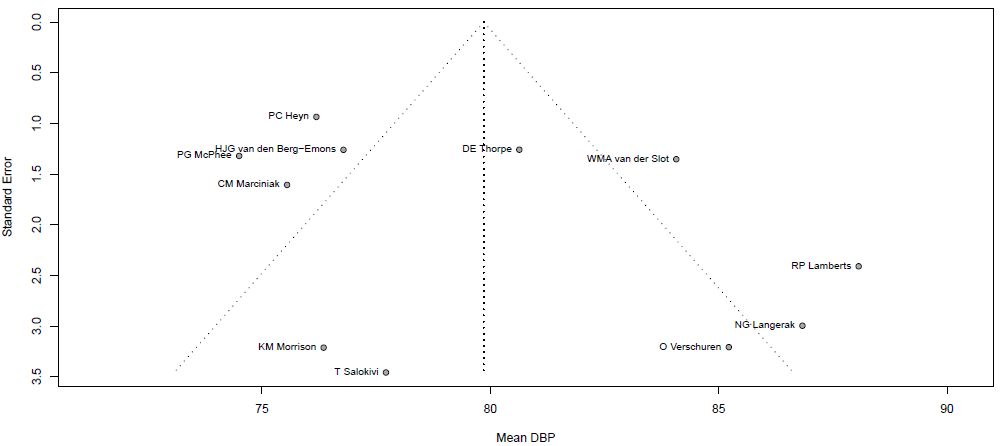


DBP=diastolic blood pressure

**S7.** Forest plots for overall prevalence of prehypertension and hypertension, and classification of blood pressure following current American Hypertension guidelines (Whelton, 2018).^[16]^ BP=blood pressure

Forest plot for prevalence of prehypertension.


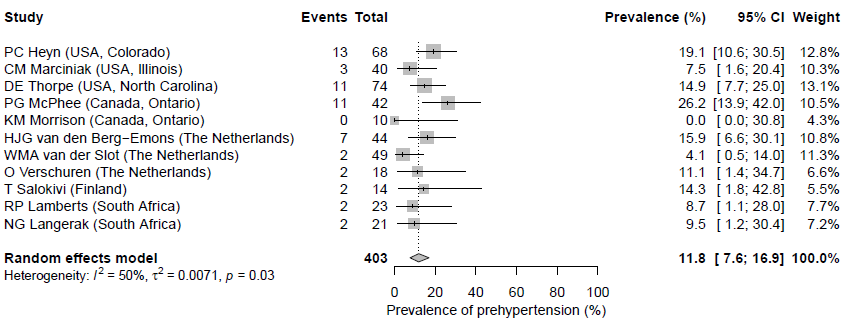
Prehypertension is defined as systolic BP 120-129 mmHg and diastolic BP <80 mmHg. Included participants N=403; participants using antihypertensive medication or with missing information on antihypertensive medication were excluded (N=41)

Forest plot for prevalence of hypertension.


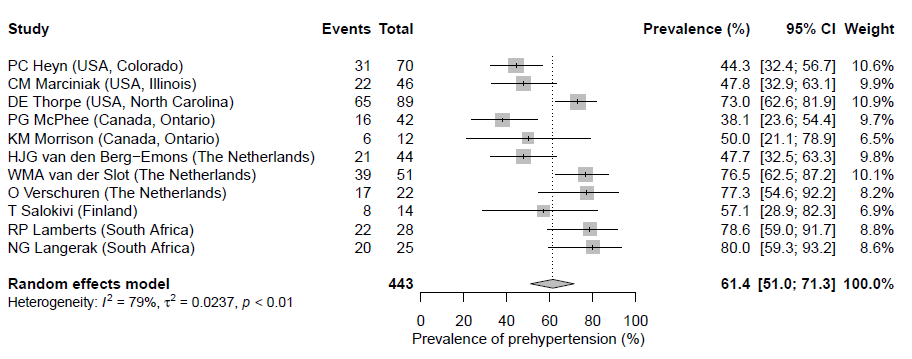


Hypertension is defined as systolic BP ≥130 mmHg or diastolic BP ≥80 mmHg or use of antihypertensive medication. Included participants N=443; participants with systolic BP < 130 mmHg and diastolic BP < 80 mmHg and missing information on antihypertensive medication were excluded (N=1)

Classification of blood pressure

| Category | Systolic blood pressure (mmHg) |  | Diastolic blood pressure (mmHg) | Number of participants | Percentage of participants |
| --- | --- | --- | --- | --- | --- |
| *Normal* | <120 | And | <80 | 121 | 30.0% |
| *Elevated* | 120-129 | And | <80 | 55 | 13.6% |
| *Hypertension stage 1* | 130-139 | or | 80-89 | 137 | 34.0% |
| *Hypertension stage 2* | 140-180 | or | 90-120 | 87 | 21.6% |
| *Hypertensive crisis* | >180 | And/or | >120 | 3 | 0.7% |

Included participants N=403; participants using antihypertensive medication or with missing information on antihypertensive medication were excluded (N=41)

**References**

1. Kokmen E, Naessens JM,Offord KP. A short test of mental status: description and preliminary results*.* *Mayo Clin Proc* 1987; 62:281-8.

2. Heyn PC, Tagawa A, Pan Z, Thomas S,Carollo JJ. Prevalence of metabolic syndrome and cardiovascular disease risk factors in adults with cerebral palsy*.* *Dev Med Child Neurol* 2019; 61:477-483.

3. Thorpe D, McMurray R, Turk M,Henderson R. Adults with Cerebral Palsy Training to Increase Overall Wellness: Project ACT NOW*.* *The Gerontologist* 2012; 52.

4. Morrison KM, Ramsingh L, Gunn E, Streiner D, Van Lieshout R, Boyle M, et al. Cardiometabolic Health in Adults Born Premature With Extremely Low Birth Weight*.* *Pediatrics* 2016; 138.

5. Salokivi T, Arvio M, Haataja L,Aaltonen S. The risk factors for metabolic syndrome among Finnish individuals with intellectual disability of 20-50 years of age living in the residential homes or more intensive care units*.* *J Intellect Disabil Res* 2015; 59:20-21.

6. Slaman J, Roebroeck M, Van Der Slot W, Twisk J, Wensink A, Stam H, et al. Can a lifestyle intervention improve physical fitness in adolescents and young adults with spastic cerebral palsy? A randomized controlled trial*.* *Arch Phys Med Rehabil* 2014; 95:1646-1655.

7. van der Slot WM, Roebroeck ME, Nieuwenhuijsen C, Bergen MP, Stam HJ, Burdorf A, et al. Cardiovascular disease risk in adults with spastic bilateral cerebral palsy*.* *J Rehabil Med* 2013; 45:866-872.

8. Bohannon RW,Smith MB. Interrater reliability of a modified Ashworth scale of muscle spasticity*.* *Phys Ther* 1987; 67:206-7.

9. Flanigan M, Gaebler-Spira D, Kocherginsky M, Garrett A,Marciniak C. Spasticity and pain in adults with cerebral palsy*.* *Dev Med Child Neurol* 2020; 62:379-385.

10. Lee K-C, Carson L, Kinnin E,Patterson V. The Ashworth Scale: A Reliable and Reproducible Method of Measuring Spasticity*.* *J Neurol Rehabil* 1989; 3:205-209.

11. Cella D, Riley W, Stone A, Rothrock N, Reeve B, Yount S, et al. The Patient-Reported Outcomes Measurement Information System (PROMIS) developed and tested its first wave of adult self-reported health outcome item banks: 2005-2008*.* *J Clin Epidemiol* 2010; 63:1179-94.

12. Wong DL,Baker CM. Smiling face as anchor for pain intensity scales*.* *Pain* 2001; 89:295-297.

13. Ware JE, Jr.,Sherbourne CD. The MOS 36-item short-form health survey (SF-36). I. Conceptual framework and item selection*.* *Med Care* 1992; 30:473-83.

14. Krupp LB, LaRocca NG, Muir-Nash J,Steinberg AD. The fatigue severity scale. Application to patients with multiple sclerosis and systemic lupus erythematosus*.* *Arch Neurol* 1989; 46:1121-3.

15. McPhee PG, Gorter JW, Cotie LM, Timmons BW, Bentley T,MacDonald MJ. Associations of non-invasive measures of arterial structure and function, and traditional indicators of cardiovascular risk in adults with cerebral palsy*.* *Atherosclerosis* 2015; 243:462-465.

16. Whelton PK, Carey RM, Aronow WS, Casey DE, Jr., Collins KJ, Dennison Himmelfarb C, et al. 2017 ACC/ AHA/ AAPA/ ABC/ ACPM/ AGS/ APhA/ ASH/ ASPC/ NMA/ PCNA Guideline for the Prevention, Detection, Evaluation, and Management of High Blood Pressure in Adults: A Report of the American College of Cardiology/American Heart Association Task Force on Clinical Practice Guidelines*.* *Hypertension* 2018; 71:e13-e115.
